# The interplay between gut bacteria and targeted therapies: implications for future cancer treatments

**DOI:** 10.1186/s10020-025-01108-6

**Published:** 2025-02-13

**Authors:** Juan He, Yu Chen, Huakan Zhao, Yongsheng Li

**Affiliations:** 1 Chongqing University Cancer Hospital, School of Medicine, Chongqing University, Chongqing, China; 2 Department of Medical Oncology, Chongqing University Cancer Hospital, 181 Hanyu Road, Shapingba District, Chongqing, 400030 China

**Keywords:** Gut bacteria, Bacterial metabolites, Anti-cancer targeted therapy, Targeted therapy molecule, Drug-resistant

## Abstract

Targeted therapy represents a form of cancer treatment that specifically focuses on molecular markers regulating the growth, division, and dissemination of cancer cells. It serves as the cornerstone of precision medicine and is associated with fewer adverse effects compared to conventional chemotherapy, thus enhancing the quality of patient survival. These make targeted therapy as a vital component of contemporary anti-cancer strategies. Although targeted therapy has achieved excellent anti-cancer results, there are still many factors affecting its efficacy. Among the numerous factors affecting anti-cancer treatment, the role of intestinal bacteria and its metabolites are becoming increasingly prominent, particularly in immunotherapy. However, their effects on anticancer targeted therapy have not been systematically reviewed. Herein, we discuss the crosstalk between gut bacteria and anticancer targeted therapies, while also highlighting potential therapeutic strategies and future research directions.

## Introduction

The intestinal tract harbors over 10 trillion bacteria that collaboratively sustain the microecological balance. The predominant intestinal bacterial communities include *Firmicutes*, *Bacteroidetes*, and *Proteobacteria*, among others (Zhao et al. 2019). These communities play a crucial role in various physiological functions, including digestion, absorption, metabolism, immune regulation, and energy conversion. The dysbiosis of intestinal bacteria is closely related to the occurrence, development, and treatment of tumors (Adak and Khan 2019). For instance, bacteria such as *Fusobacterium nucleatum, Escherichia coli,* or *Bacteroides fragilis* are implicated in several pathological processes, including colorectal carcinogenesis, infiltration, and metastasis (Qiu et al. 2022; Meng et al. 2018). Furthermore, gut bacteria-derived metabolites are also closely associated with carcinogenesis, cancer progression and anti-cancer therapy, including short-chain fatty acids (SCFAs), secondary bile acids (SBAs), indole derivatives, and trimethylamine-N-oxide (TMAO), etc. Notably, butyrate can inhibit histone deacetylase (HDAC), inducing apoptosis in cancer cells and suppressing their proliferation. Propionate reduces the risk of colorectal cancer by inhibiting the inflammatory response in cancer cells (Tan et al. 2014; Wang et al. 2020). Lithocholic acid can activate the NF-κB signaling pathway, promoting tumor cell proliferation and metastasis (Chiang and Ferrell 2020). Meanwhile, indole derivatives modulate the aryl hydrocarbon receptor (AHR) pathway, influencing the immune system’s surveillance of cancer and thereby affecting tumor growth and the efficacy of immunotherapy (Fu et al. 2023).

Targeted therapies represent a complex and continually evolving domain that plays a pivotal role in cancer treatment. These therapies are widely utilized across various cancer types, including breast, lung, colorectal, melanoma, and certain leukemias (Guo et al. 2021; Tiwari et al. 2018). The essence of these strategies lies in the identification and selection of specific molecular targets, such as the vascular endothelial growth factor receptor (VEGFR) (Sitohy et al. 2012), epidermal growth factor receptor (EGFR) (Zhou et al. 2021), human epidermal growth factor receptor 2 (HER2) (Meric-Bernstam et al. 2019), insulin-like growth factor 1 receptor (IGF-1R) (Tzanakakis et al. 2021). These targets are critical molecules that regulate cancer cell survival, growth, and metastasis. By focusing on these specific targets, targeted therapies can more precisely attack cancer cells. This approach offers significant advantages over traditional chemotherapy, including the ability to tailor treatment plans to the patient’s unique genetic characteristics and disease status. Such personalization enhances specificity, minimizes damage to surrounding healthy cells, and consequently reduces side effects while improving patient outcomes. However, it is important to acknowledge that targeted therapy also faces numerous challenges. Despite the approval and positive efficacy of several drugs by the U.S. Food and Drug Administration (FDA), such as rituximab, trastuzumab, cetuximab, and bevacizumab (Zhong et al. 2021), limitations persist in their clinical application. Factors affecting the efficacy of targeted therapy include drug resistance from long-term use, adverse reactions, differences in genetic mutations, tumor heterogeneity, and the complexity of the tumor microenvironment. In addition, drug absorption, distribution, metabolism, and individual differences can also lead to variations in treatment outcomes. These factors collectively affect the efficacy of targeted therapy, adding to the complexity of treatment (Lim and Ma 2019). However, the impact of the gut bacteria on targeted therapy has not been comprehensively reviewed.

Recent research has increasingly recognized the intricate relationship between intestinal bacteria and the effectiveness of anti-cancer targeted therapies. These bacteria can modulate the metabolic pathways of antitumor drugs by influencing critical molecules and pathways, thereby affecting both the efficacy and side effects of these treatments. Specifically, molecular targeted therapeutic drugs, such as inhibitors of VEGF, EGFR, and HER2, play a pivotal role in the management of malignant tumors, with their effectiveness being closely tied to the composition of the intestinal microbiota (Huang et al. 2019). Furthermore, the interactions between microbes and tumors may contribute to the development of drug resistance, presenting a significant challenge in cancer treatment (Yu et al. 2017). Therefore, in the context of antitumor targeted therapy, it is essential to consider not only the characteristics of tumor cells but also to enhance our understanding of the gut microbiome and its metabolic interactions in individual patients. This approach will facilitate the development of personalized and effective tumor-targeted therapeutic strategies, ultimately improving therapeutic outcomes and quality of life for patients.

This review aims to summarized the crosstalk between intestinal bacteria and anti-cancer targeted therapies, and to explore the promising therapeutic strategies related to these gut bacteria and its metabolites, thereby opening new avenues for anticancer targeted therapy.

## Impact of anticancer targeted therapy on gut bacteria

**Fig. 1:**
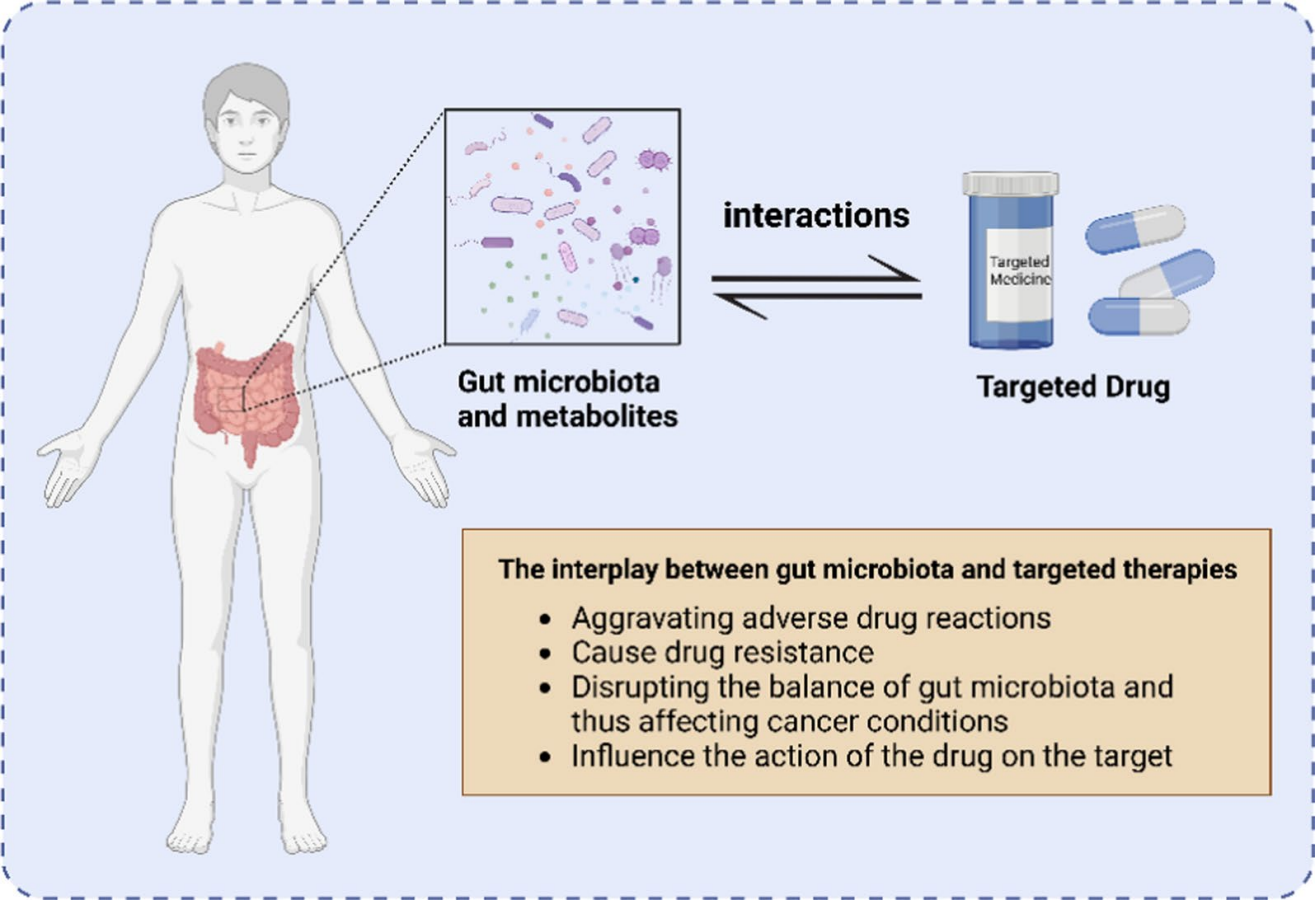
The potential interaction between gut microbiota and targeted drugs: In cancer patients, taking targeted drugs may lead to gut microbiota imbalance, which could exacerbate drug side effects or affect cancer progression. Gut microbiota and its metabolites can contribute to tumor resistance to targeted drugs. Additionally, gut microbiota and its metabolites may also impact drug targets

The gut microbiota represents one of the most crucial microbial ecosystems within the human body, comprising approximately 1000 to 1150 bacterial species (Qiu et al. 2022; Wong and Yu 2023). These microorganisms play a vital role in maintaining the homeostasis of the digestive system, contributing not only to the digestion and absorption of nutrients but also to the preservation of the intestinal barrier, the regulation of the immune system, and the defense against pathogenic invasions (Perler et al. 2023). Furthermore, the homeostatic state of gut bacteria can be influenced by various factors, including diet, lifestyle, genetic predispositions, and pharmacological treatments (Perler et al. 2023; Park et al. 2022). In cancer patients undergoing antitumor targeted therapies, significant alterations in gut bacteria occur, which in turn impact the patients’ intestinal health and treatment efficacy (Chen et al. 2021). Tumor cells are not the specific targeting of some anti-cancer targeted drugs which can also disrupt the normal gut bacteria, leading to changes in bacterial composition and a reduction in microbial diversity (Wong and Yu 2023; Helmink et al. 2019). This disruption in the intestinal microbial community is termed dysbiosis (Tojo et al. 2014), which may result in compromised intestinal barrier function, heightened inflammation, and immune system dysfunction, ultimately affecting the effectiveness of tumor therapies and the quality of life for patients (Fig. 1).

Numerous clinical studies have identified diarrhea as one of the most prevalent adverse effects associated with targeted therapies, which is closely linked to the dysbiosis of gut bacteria. For instance, in patients with metastatic renal cell carcinoma (mRCC) undergoing treatment with sunitinib, those experiencing diarrhea exhibited a significantly higher abundance of *Bacteroides*, a type of mucus-degrading bacteria, alongside a reduced presence of butyrate-producing bacteria, such as *Faecalibacterium*, *Oscillospira*, *Ruminococcaceae*, *Eubacterium*, and *Coriobacteriaceae*, compared to their counterparts without diarrhea (Su et al. 2021). Similarly, in patients with hepatocellular carcinoma treated with lenvatinib, the diarrhea group demonstrated a significantly greater relative abundance of *Bacteroidales*, including *Parabacteroides* and *Prevotella*, compared to the non-diarrhea group (Inukai et al. 2023). Furthermore, among mRCC patients receiving VEGF-tyrosine kinase inhibitor (TKI) therapy, those with diarrhea exhibited elevated levels of *Bacteroides spp.* and diminished levels of *Prevotella spp* (Secombe et al. 2020). In a rat model, diarrhea induced by small molecule receptor TKIs such as lapatinib resulted in decreased intestinal microbial diversity, with a notable increase in Proteobacteria, particularly within the *Betaproteobacteria* class (Mayo et al. 2020). These findings underscore a direct correlation between gut microbiota dysbiosis and anti-cancer targeted drug-drived adverse effects, suggesting a potential strategy for targeting these gut bacteria to mitigate the drug side effects.

In addition to drug adverse effects, anti-cancer targeted drug-related gut microbiota dysbiosis can also reflect the anti-cancer efficacy. The 16S rRNA sequencing of intestinal flora in patients with recurrent malignant gliomas treated with bevacizumab in combination with temozolomide revealed significant differences in the composition and abundance of fecal bacteria between those receiving the combination therapy and those treated with temozolomide alone. Notably, the increased abundance of *Firmicutes* and *Bacteroidetes* may reflect patient responses to bevacizumab, indicating a potential role of gut flora in tumor therapy outcomes (Zhu and Su 2022). Additionally, in patients with metastatic colorectal cancer undergoing first-line treatment with anti-VEGF agents (bevacizumab) or anti-EGFR agents (cetuximab), the presence of *K. quasipneumoniae*, *Limosilactobacillus mucosae*, *Lactobacillus species*, *Bifidobacterium dentium*, *Bifidobacterium breve*, *Bifidobacterium bifidum*, *Veillonella atypica*, *Veillonella dispar*, *Veillonella nakazawae*, and *Veillonella S12025-13* was significantly higher in the progressive disease (PD) group compared to the partial response (PR) group. Conversely, *F. nucleatum*, *Phascolarctobacterium sp*. *Marseille-Q4147*, *Prevotella dentalis*, and *Prevotella copri* were significantly more abundant in the PR group than in the PD group (Chen et al. 2022). Furthermore, a higher diversity of intestinal flora was associated with a more favorable therapeutic effect, although it was not linked to the occurrence of diarrhea. These findings suggest that the therapeutic effects of bevacizumab and cetuximab may characterized by the alterations of intestinal bacteria, some of which potentially playing an auxiliary role in anti-cancer treatment, thereby highlighting the possibility of targeting these intestinal bacteria in anti-cancer targeted therapies.

In conclusion, the balance of gut flora is essential for human health and disease management. Alterations in gut flora during antitumor-targeted therapies can lead to adverse effects or influence therapeutic outcomes in cancer patients. This suggests that the composition of gut bacteria may significantly impact the efficacy of tumor-targeted treatments.

## Impact of intestinal flora on targeted therapies

Targeted therapies are designed to enhance therapeutic efficacy while minimizing damage to normal cells by accurately identifying and targeting specific molecular entities associated with disease. The most widely used signal molecules in anti-tumor targeted therapy are tyrosine kinases, angiogenic factors, signaling molecules, and the proteins involved in cell cycle.

Since Folkman introduced the concept of tumor neoangiogenesis in the 1970s, considerable progress has been made in the research of inhibitors that target this process (Liu et al. 2023a). Antibodies or receptor tyrosine kinase (RTKs) inhibitors that target VEGF, VEGFR, FGFR, EGFR, or other neoangiogenesis-related molecules represent a significant area of the researches in anti-cancer targeted therapies. The targets of these RTKs include various molecules closely related to cell proliferation, survival, and angiogenesis, such as HER2, PDGF, VEGFR, FGFR, and IGF-1R. Moreover, over 20 receptor and non-receptor tyrosine kinases from various families have been investigated for antitumor drug screening, such as Herceptin and Pertuzumab targeting HER2, Bevacizumab targeting VEGF, Afatinib and Gefitinib targeting EGFR, and Sorafenib and Lenvatinib targeting multiple RTKs (Tomuleasa et al. 2024). However, these drugs often encounter challenges related to drug resistance and side effects in clinical applications. The stimuli generated by the binding of growth factors and other external cellular signals to their specific receptors are transmitted to cells through a network of signaling pathways, which together form a complex intracellular signal transduction system that regulates cell proliferation and differentiation. Collectively, these pathways form a complex intracellular signal transduction system that regulates cell proliferation and differentiation. In targeted therapy, these signaling pathways serve as critical targets that significantly influence tumorigenesis and progression. The PI3K/AKT/mTOR signaling pathway is composed of phosphatidylinositol 3-kinase (PI3K) and its downstream components, including protein kinase B (PKB/AKT) and the mechanistic target of rapamycin (mTOR). Currently, the FDA has approved four PI3K inhibitors for the treatment of lymphoma: the broad-spectrum PI3K inhibitor Copanlisib, the selective PI3Kδ inhibitor Idelalisib, the dual PI3Kδ/γ inhibitor Duvelisib, and the PI3Kδ/CK1ε inhibitor Umbralisib. Additionally, mTOR inhibitors such as Everolimus, Rapamycin, and Ridaforolimus are utilized in clinical settings (Glaviano et al. 2023). Oncogenic mutations in the RAS/MAPK signaling pathway members RAS, RAF, and MEK lead to the sustained activation of downstream signaling pathways, making them important drivers of various tumors and key targets for cancer treatment. Drugs targeting the RAS/MAPK pathway include Adagrasib and Sotorasib (Bahar et al. 2023). The signal transducer and activator of transcription (STAT) family was initially identified as DNA-binding proteins capable of mediating interferon-dependent gene expression and can also mediate various intracellular signaling pathways. The STAT family operates downstream of the PI3K/AKT/mTOR, Ras/MAPK, and JAK pathways. The JAK/STAT pathway plays a crucial role in cell proliferation, differentiation, and immune responses, and its aberrant activation is closely associated with the development of various cancers. Current research targeting the JAK/STAT pathway includes several small molecule inhibitors and monoclonal antibodies, such as the JAK inhibitors Ruxolitinib, Tofacitinib, and Baricitinib. These agents inhibit the activity of JAK enzymes, reducing the phosphorylation of STAT proteins, thereby interfering with signaling pathways and helping to slow tumor progression while improving patient prognosis (Alzahrani 2019).

The intestinal bacteria or its metabolites, such as LPS, secondary bile acids, SCFAs, TMAO, and indole derivatives, may interfere with the effectiveness of anti-tumor targeted therapies by affecting the mentioned targets, thereby significantly impacting the overall therapeutic efficacy and patient prognosis. Therefore, understanding the interactions between the intestinal bacteria and these signaling pathways is crucial for optimizing anti-tumor targeted treatment strategies.

### Influence of intestinal bacteria on signaling targets for anti-cancer targeted therapy

The intestinal flora significantly influences cancer development and therapy. Specific bacteria play a crucial role in the efficacy of anticancer targeted therapies by directly or indirectly modulating signaling pathways associated with tumor proliferation, survival, and migration.

It is well established that RTKs contribute to oncogenesis by promoting tumor cell proliferation and metastasis. Consequently, numerous studies have explored the impact of bacterial interventions on tumor progression through their effects on RTKs. For instance, the expression levels of both ErbB2 (HER2) and ErbB3 (HER3), the members of the RTK family, were found to be elevated in colorectal cancer (CRC) cells when exposed to *Listeria monocytogenes* supernatant (Oliveira et al. 2005). Additionally, another study revealed that the surface protein InlB of *L. monocytogenes* facilitates bacterial entry into mammalian cells and induces the tyrosine phosphorylation of mesenchymal to epithelial transition factor (MET), thereby activating downstream signaling pathways and biological processes, including Gab1 and Cbl phosphorylation and epithelial cell scattering (Shen et al. 2000). Consequently, *L. monocytogenes* promotes the proliferation and migration of colorectal cancer cells by upregulating the expression of RTK family members ErbB2 and ErbB3, and inducing the tyrosine phosphorylation of Met. Furthermore, *Fusobacterium nucleatum*, typically present in low abundance in the intestines of healthy individuals, exhibits increased abundance in patients with CRC, breast cancer, or ulcerative colitis (Alon-Maimon et al. 2022). *F. nucleatum* has been shown to induce alterations in the expression of molecular markers associated with epithelial-mesenchymal transition (EMT) through the activation of the EGFR and its downstream signaling pathways. This activation enhances the invasive and migratory capabilities of CRC and colorectal adenocarcinoma (CAC) cells, thereby accelerating tumor growth and increasing tumorigenic potential (Yu et al. 2020). Zhang et al. also found that *F. nucleatum* can bind to receptors expressed on host cells, activating the oncogenic PI3K/AKT/NF-κB cascade. This promotes the expression of metalloproteinases MMP-2/9, thereby enhancing the development and progression of colorectal cancer (Zhang et al. 2024). Consequently, *F. nucleatum* may impede the therapeutic efficacy of RTK-targeted treatments by activating RTKs and PI3K/AKT/NF-κB. Humans can alter the balance of the gut microbiota by consuming fermented foods or supplementing with relevant probiotics. Through these methods, the probiotic *Bacillus polyfermentans* can enter the gut and establish residence. *B. polyfermentans* has demonstrated the ability to reduce tumor size, inhibit tumor cell proliferation, and suppress colony formation. A study utilizing conditioned media from *B. polyfermentans* suggests that a soluble bacterial compound may function as a biological anticancer agent. The potential molecular mechanisms underlying the reduction in tumor growth may involve decreased expression of ErbB2 and ErbB3, as well as the inhibition of the cell cycle regulator cytosolic protein D. The findings indicate that enhancing the intestinal enrichment of *B. polyfermentans* could yield synergistic antitumor effects when combined with RTK-targeted therapies (Ma et al. 2010). Crohn’s disease (CD) is an inflammatory bowel disease. It has been reported that patients with long-term CD or ulcerative colitis (UC) have a 2–3 times higher risk of developing CRC compared to the general population. Sanda Minmoun et al. demonstrated in human intestinal epithelial cells and transgenic mouse models that adherent-invasive *Escherichia coli* (AICE) could contribute to the pathogenesis of CD by increasing VEGF/VEGFR-2 signaling and interleukin-8 (IL-8) production (Mimouna et al. 2011).

*Porphyromonas gingivalis*, recognized for its association with periodontitis, has been found to be enriched in fecal and mucosal samples from CRC patients. This bacterium can activate the RAS/RAF/MEK/ERK signaling pathway through its gingival protease, leading to the downstream phosphorylation of MEK1/2 and ERK1/2. Consequently, it can be hypothesized that *P. gingivalis* may promote the proliferation of CRC cells by modulating the MAPK/ERK signaling pathway (Mu et al. 2020). Furthermore, *P. gingivalis* activates the ERK1/2-Ets1, p38/HSP27, and PAR2/NF-κB pathways by inducing the expression of proto-matrix metalloproteinase-9 in oral squamous cell carcinoma (OSCC) cells, and thereby facilitating cell invasion and metastasis (Inaba et al. 2014). Thus, *P. gingivalis* may possess the potential to inhibit antitumor therapies that target the MEK/ERK pathway. The anaerobic digestive streptococcus *Peptostreptococcus anaerobius* is selectively enriched in the fecal and mucosal microbiota of CRC patients. Research has demonstrated that the surface protein PCWBR2 of *P. anaerobius* directly interacts with integrin α2/β1 located on the surface of CRC tumor cells. This interaction initiates an oncogenic cascade involving PI3K/AKT/FAK, which promotes tumor cell proliferation and contributes to the development of CRC (Long et al. 2019). Therefore, P. anaerobius can promote cancer development and progression by activating the PI3K/AKT/FAK pathway (Table 1).

**Table 1:** The interaction between gut bacteria and anti-cancer targets

Gut bacteria	Affected targets/pathways	Disease	Effect on therapy	References
*Listeria monocytogenes*	MET, HER2, HER3	Colorectal cancer	Promote HER2/3 expression and activate MET phosphorylation	Oliveira et al. (2005), Shen et al. (2000)
*Fusobacterium nucleatum*	VEGF, EGFR, PI3K/AKT/NF-κB	Colorectal cancer	Activate the EGER and PI3K/AKT/NF-κB pathway	Alon-Maimon et al. (2022), Yu et al. (2017), Zhang et al. (2024)
*Bacillus polyfermentans*	HER2, HER3	Colorectal cancer	Decreased expression of HER2/3	Ma et al. (2010)
*Porphyromonas gingivalis*	MEK, ERK	Colorectal cancer	Activate the MEK/ERK pathway	Mu et al. (2020), Inaba et al. (2014)
*Peptostreptococcus anaerobius*	PI3K/AKT	Oral squamous cell carcinoma, colorectal cancer	Activate the PI3K/AKT pathway	Long et al. (2019)

### Influence of the intestinal bacteria-derived metabolites on signaling targets for targeted therapy

#### LPS

**Fig. 2:**
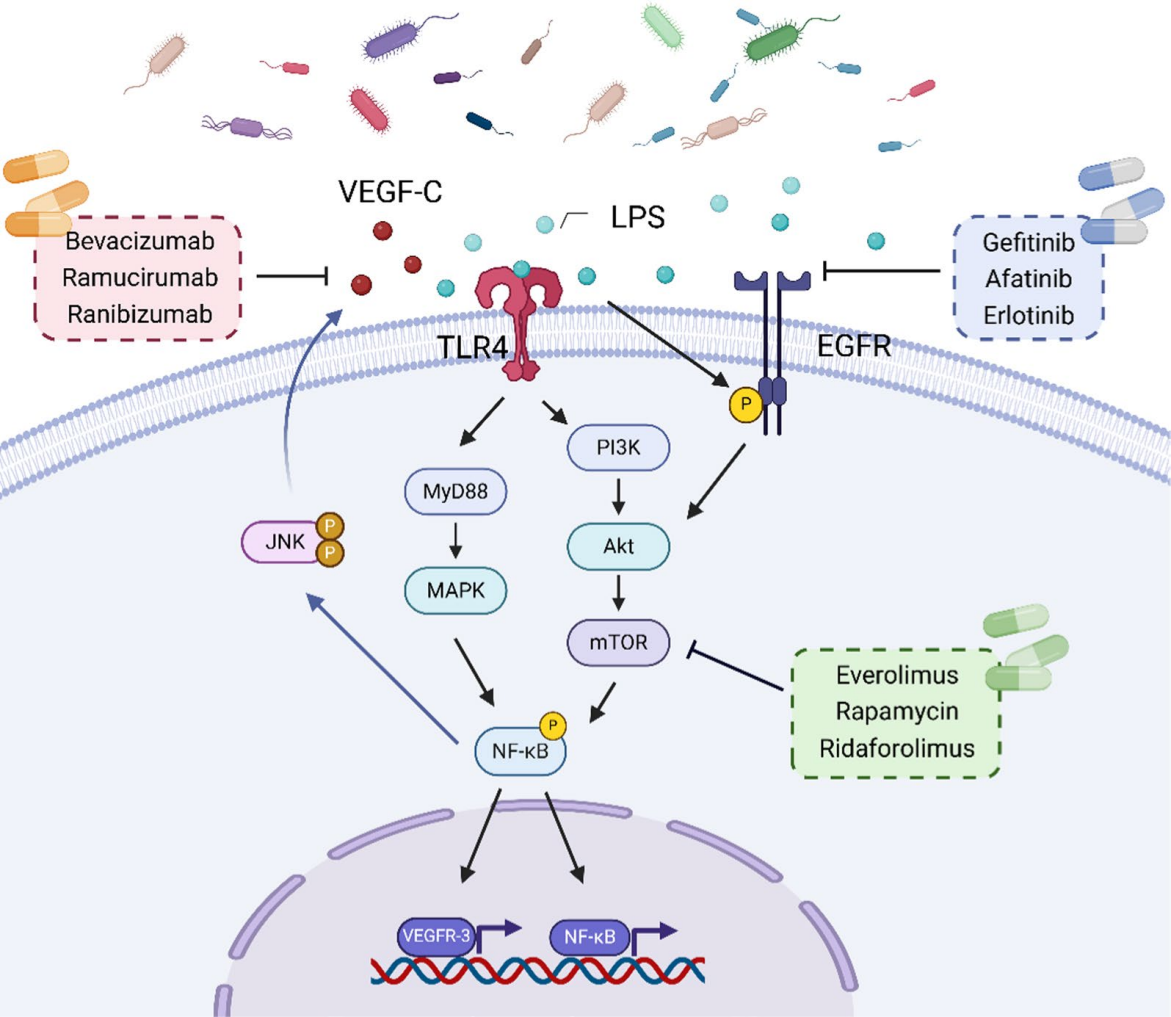
The relationship between LPS and various targets: LPS binds to TLR4, activating the PI3K/AKT/mTOR and MyD88/MAPK signaling pathways, which in turn promotes the activation and transcription of NF-κB, as well as the transcription of VEGFR-3. LPS also enhances the secretion of VEGF-C by promoting the activation of the NF-κB/JNK pathway

Lipopolysaccharide (LPS) is a significant component of the cell wall of gram-negative (G^−^) bacteria, comprising a complex of lipids and polysaccharides (Rathinam et al. 2019). It consists of three distinct parts: lipid A, the core polysaccharide, and the O-specific chain. Among these, lipid A serves as the fatty component of LPS, is central to its bioactivity, and is identified as the primary toxic component (Wang and Quinn 2010). Following bacterial death, LPS is cleaved and released into both the intestinal environment and the bloodstream (Banaszak et al. 2023). Recent studies have revealed a strong correlation between gut bacteria-drived LPS and anti-cancer targeted therapy-related molecules (Fig. 2).

VEGFR-3, a member of RTKs family, is involved in promoting tumor invasion andmetastasis, tumor neoangiogenesis, and tumor resistance to chemotherapy. Zhu et al*.* (2016a) demonstrated that LPS can bind to Toll-like receptor 4 (TLR4) on the surface of CRC cells, leading to the activation of the NF-κB signaling pathway. This activation subsequently enhances the transcription of VEGFR-3, thereby promoting the migration and invasion of CRC. Furthermore, EREG, a multifunctional cytokine belonging to the epidermal growth factor (EGF) family, plays a critical role in various physiological and pathological processes by interaction with its receptor EGFR. A study found that LPS stimulation in mice increased EREG expression in tumor tissues, which enhanced tumor angiogenesis via IL-8 signaling and promoted the migration and invasion of EGFR-positive hepatocellular carcinoma (HCC) cells (Kubo et al. 2024). Nakanaga et al. (2007) reported that LPS could activate EGFR through the Duox1-TACE-TGF-α signaling pathway. Additionally, Tao et al*.* discovered that LPS stimulation in mice resulted in increased EGFR expression and activation of the downstream AKT and ERK1/2 pathways. Notably, the activation of AKT and ERK1/2 was found to enhance the functionality of the transcription factor NF-κB (Tao et al. 2019). Moreover, Zhu et al. (2016b) identified that LPS can increase VEGF-C secretion in colorectal cancer cells through the TLR4/NF-κB/JNK signaling pathway, thereby promoting both the motility of colorectal cancer cells and lymphangiogenesis.

mTOR is a highly conserved serine/threonine protein kinase that plays a crucial role in tumorigenesis and cancer development through the PI3K/AKT/mTOR signaling pathway. Consequently, mTOR has emerged as a significant target for anti-cancer targeted therapy. One study indicated that LPS activates mTOR and its downstream effector NF-κB by promoting TLR4/MyD88/MAPK signaling (Zhou et al. 2018). Additionally, Li et al. (2018) demonstrated that LPS promotes the mTOR/STAT3 pathway. Furthermore, He et al. (2009), Hu et al. (2020) showed that LPS activates primary cultured mouse lung cells and the PI3K/AKT/mTOR signaling pathway in human lung fibroblast MRC-5 cells via its receptor TLR4. Collectively, these findings suggest that bacterial LPS enhances RTKs and the PI3K/AKT/mTOR signaling pathway, which may impede antitumor therapies targeting RTKs and PI3K/AKT/mTOR (Fig. 2).

#### Secondary bile acids

Bile acids consist of primary bile acids and secondary bile acids, the former of which is produced by the conversion of cholesterol in the mammalian liver, and the latter of which is produced by intestinal bacteria using the conversion of primary bile acids transported via enterohepatic axis (Wahlström et al. 2016). Secondary bile acids, such as deoxycholic acid (DCA), lithocholic acid (LCA) and ursodeoxycholic acid (UDCA) (Guzior and Quinn 2021), play a crucial role not only in the digestion and absorption of lipids but also in maintaining the homeostasis of the bile acid pool, regulating the composition of intestinal microbiota, and activating specific receptors, including the FXR and GPCRs. These processes significantly influence energy metabolism and immunomodulation (Guzior and Quinn 2021; Winston and Theriot 2020). Many gut microbes are involved in the synthesis of secondary bile acids. *Clostridium* (clusters XIVa and XI) and *Eubacterium* can synthesize DCA and LCA. Fewer bacteria are known to produce UDCA, but *Eubacterium lentum* has been identified as capable of converting other bile acids into UDCA. Furthermore, other gut bacteria, such as *Bacteroides*, *Faecalibacterium*, *Bilophila*, and *Ruminococcus*, are also capable of producing secondary bile acids, thereby regulating specific targets and influencing the progression of various diseases (Cai et al. 2022).

**Fig. 3:**
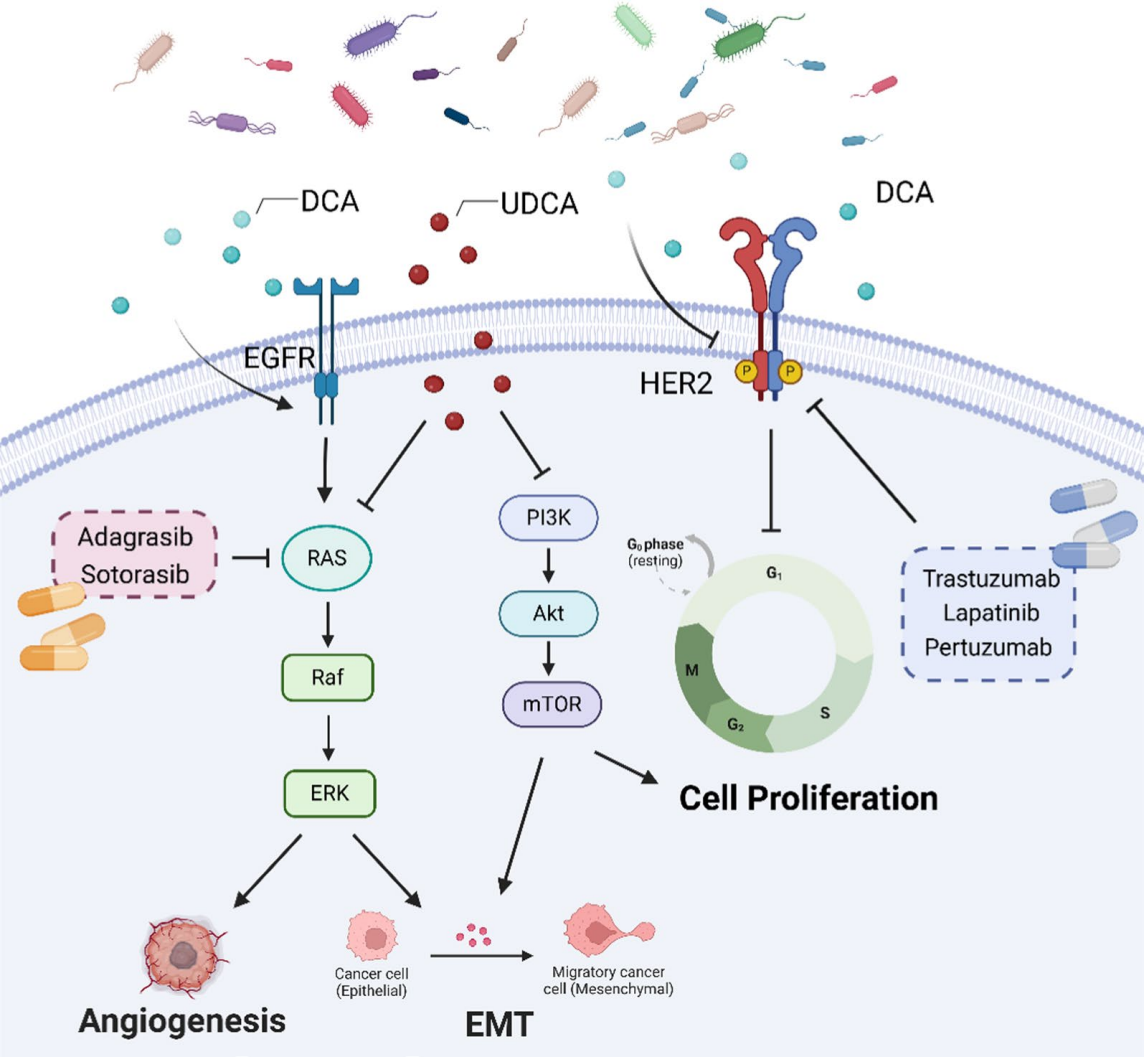
The relationship between secondary bile acids and their targets: UDCA can inhibit the PI3K/AKT/mTOR and RAS/Raf/ERK signaling pathways, thereby suppressing tumor proliferation, EMT, and angiogenesis. In contrast, DCA can promote the activation of EGFR and its downstream pathways, facilitating the development of tumor cells. However, DCA also has cancer-inhibiting effects. DCA can inhibit the HER2 target, thereby suppressing the proliferation of cancer cells

Secondary bile acids have been reported to regulate the expression of RTKs (Yoon et al. 2002; Werneburg et al. 2003). Qiao et al. (2001) found that DCA directly induced rapid phosphorylation of the EGFR in rat HCC cells. In HM3 colon adenocarcinoma cells, DCA activates the EGFR/Ras/Raf-1/ERK signaling pathway, resulting in the upregulation of mucin 2 (MUC2). Notably, the specific EGFR inhibitor AG1478 effectively blocked DCA-induced MUC2 upregulation (Lee et al. 2010). Furthermore, DCA significantly enhances the local spatial aggregation of phosphatidic acid (PA), promoting co-localization of PA with EGFR, which facilitates EGFR dimerization and stimulates EGFR-MAPK signaling in hepatocytes and gastrointestinal cells, including Barrett’s-associated esophageal cells, gastric cancer cells, cholangiocarcinoma cells, and colon cancer cells (Fang et al. 2021). These studies have found that DCA can promote the activation of EGFR, thereby facilitating the development and progression of cancer. Similar studies have also found that high-fat diet (HFD) not only promotes vasculogenic mimicry (VM) but also disrupts the intestinal bacteria. This disruption results in an increase in the intestinal microbial metabolite DCA, which drives the processes of EMT and VM formation by activating VEGFR2 signaling, thereby contributing to the development of colorectal cancer. Conversely, DCA may directly affect the HER2 target and exert an anti-cancer effect. Wang et al. (2022) discovered that DCA, when metabolized by *Clostridium*-specific metabolites, facilitates the transition of HER2-positive breast cancer cells from the G0/G1 phase into the S-phase, thereby inhibiting cell proliferation and hindering breast cancer progression. However, this effect does not extend to triple-negative breast cancer. Although the precise mechanism underlying these findings remains unclear, they enhance our understanding of the influence of intestinal flora on HER2-positive breast cancer. These studies suggest that DCA may exert an inhibitory effect on antitumor therapies that target RTKs and HER2 (Fig. 3).

Ursodeoxycholic acid (UDCA) belongs to the family of hydrophilic secondary bile acids and has been shown to prevent apoptosis induced by various stress stimuli (Liu et al. 2015). Several studies indicate that UDCA may have a role in tumor prevention. For instance, UDCA not only can inhibit the activation of the EGFR-ERK pathway, thereby suppressing the proliferation of cholangiocarcinoma cells, but also can inhibit the expression of PI3K and AKT, providing further evidence for its anti-tumor effects in cholangiocarcinoma cells (Lee et al. 2021). Rebecca suggested that UDCA promotes an interaction between EGFR and caveolin-1, which enhances the suppression of MAP kinase activity and cell growth mediated by UDCA (Feldman and Martinez 2009). Another study found that UDCA can also inhibit IGF-1-induced activation of AKT and ERK, which are key pathways involved in regulating the proliferation of cholangiocarcinoma cells (Lee et al. 2019). In HCC, UDCA is found to enhance the sorafenib-induced downregulation of phospho-STAT3, thus increasing the inhibitory effect on tumorgrowth (Lee et al. 2018). Collectively, these studies suggest that UDCA has the potential to enhance anti-tumor therapies targeting RTKs, STAT and the PI3K/AKT pathway (Fig. 3).

#### Short-chain fatty acids

Short-chain fatty acids (SCFAs), such as acetate, propionate, and butyrate, are generated by specific intestinal microorganisms through the fermentation of otherwise indigestible dietary fibers (Koh et al. 2016). The levels of SCFAs in fecal and plasma samples are positively correlated with both the abundance of SCFA-producing microbiota in the gut and dietary fiber intake (Tan et al. 2014; Koh et al. 2016). Butyrate-producing bacteria primarily consist of bacteria from the families *Ruminalococcaceae* and *Lachnospiraceae*, as well as species such as *Anaerobutyricum hallii* and *Anaerostipes spp.* (Martin-Gallausiaux et al. 2021; Hou et al. 2022; Trompette et al. 2022) In contrast, acetate and propionate are predominantly produced by *Bifidobacterium* and mucin-degrading bacteria, such as *Akkermansia muciniphila* (Vos et al. 2022).

Butyrate, a naturally occurring metabolite of gut bacteria, is a four-carbon SCFA that plays a significant role in health and the aging process (Martin-Gallausiaux et al. 2021; Stoeva et al. 2021). Butyrate is believed to play a significant role in the occurrence (O’Keefe 2016), development, and treatment of tumors. As the primary energy source for colonic epithelial cells, butyrate also regulates gene expression by inhibiting histone deacetylase (HDAC) (Li et al. 2021a; Bridgeman et al. 2022), thereby affecting cell proliferation, differentiation, and apoptosis. Additionally, butyrate can act as a local signaling molecule involved in cellular metabolism (Tian et al. 2023), regulating tumor cells and playing an important role in modulating the tumor microenvironment and immune response (Liu et al. 2018; Stilling et al. 2016). Butyrate has been demonstrated to downregulate VEGF expression in a dose-dependent manner (Sawa et al. 2002; Pellizzaro et al. 2002). Furthermore, butyrate can activate Tyrosine Phosphatase to dephosphorylate Sp1, which inhibits the binding of Sp1 to the VEGF promoter, and leads to reduced VEGF expression and angiogenesis, subsequently diminishing tumor metastasis (Prasanna Kumar et al. 2008). Other studies have found that butyrate can inhibit the overphosph orylation of JAK2/STAT3 and eliminate the increase in p-JAK2 and p-STAT3 protein levels induced by MPP (Ji et al. 2023). EHLJ7 is a quaternary coptisine derivative. Tang et al. (2019) demonstrated that EHLJ7 can promote the production of SCFAs, particularly butyrate. EHLJ7, in conjunction with butyrate, synergistically inhibits the JAK2/STAT3/SOCS1 pathway. These observations suggest that butyrate can inhibit the VEGF and JAK2/STAT3 signaling pathway, thereby exerting anti-tumor effects. Interestingly, Dariushnejad et al. (2023) found that sodium butyrate (NaB) at a concentration of 200 mg/kg increased the expression of VEGFR2 and several angiogenesis-related proteins, including NOx, AKT, ERK1/2, and VEGF-A. Overall, butyrate exerts different effects in various types of cancers.

Neuropilin-1 (NRP-1) is a transmembrane glycoprotein that acts as a co-receptor for the tubulin VEGF family. NRP-1 binds or regulates a number of other extracellular ligands and thus participates in a variety of physiological and pathological processes, such as tumorigenesis and progression, angiogenesis, and immune response. These suggest that NRP-1 is a potential therapeutic target (Liu et al. 2020). It has been demonstrated that butyrate down-regulates NRP-1 and VEGF at the mRNA and protein level in CRC cell lines (Yu et al. 2010). Another study also found that butyrate can downregulate the expression of NRP-1 in adenoma cell lines (Yu et al. 2011). These studies suggest that butyrate can target NRP-1 to exert anti-tumor effects.

**Fig. 4:**
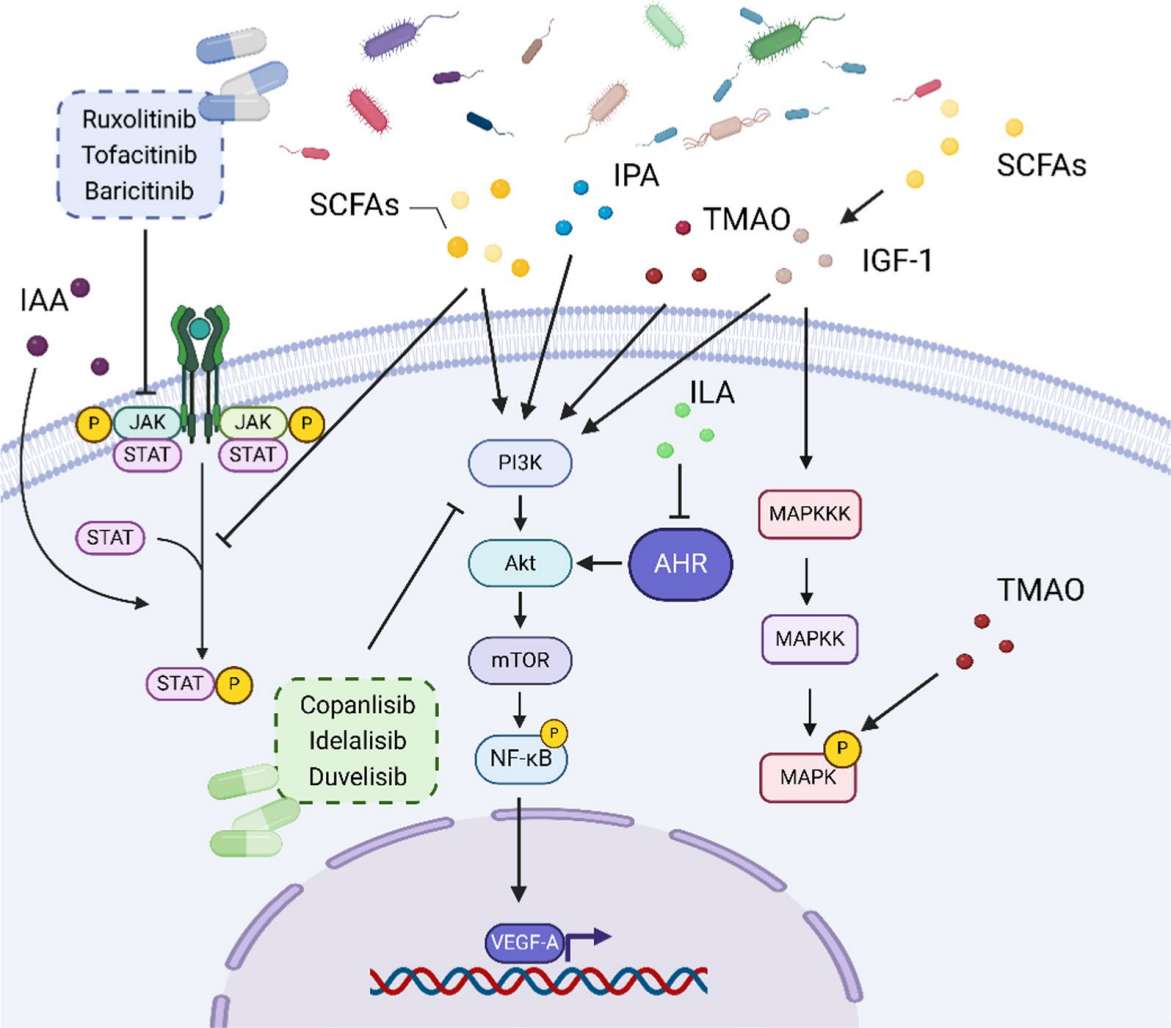
The relationship between SCFAs, TMAO, and indole derivatives with various targets: Different gut microbiota metabolites have distinct effects on cancer, influencing different targets in various ways. SCFAs, TMAO, and the indole derivative IPA can activate the PI3K/AKT/mTOR/NF-κB pathway, thereby promoting VEGF-A expression. SCFAs can stimulate IGF-1 production, which in turn activates the PI3K/AKT/mTOR and MAPK pathways. SCFAs can also inhibit the phosphorylation of STAT. However, the indole derivative IAA can promote STAT phosphorylation. TMAO can enhance MAPK phosphorylation. ILA can inhibit AHR activity, thereby suppressing its downstream AKT

Extensive research has revealed that that SCFAs play a regulatory role in the expression of human insulin-like growth factor-1 (IGF-1), a crucial hormone for bodily growth, yet a detrimental factor in prostate cancer. Matsushita et al. (2021) demonstrated that SCFA-induced IGF-1 production from gut bacteria influences prostate cancer growth by activating local prostate MAPK and PI3K signaling pathways, thereby suggesting the existence of a gut microbiota-IGF-1-prostate axis. Additionally, Yan et al. (2016) found that SCFAs elevate systemic IGF-1 levels, which subsequently contribute to bone formation and growth. Overall, these studies suggest that SCFAs may influence disease progression by regulating the expression of VEGF, JAK2/STAT3, NRP-1, and IGF-1 (Fig. 4).

#### TMAO

TMAO is a significant metabolite produced by both intestinal flora and the liver (Gatarek and Kaluzna-Czaplinska 2021). Initially, trimethylamine (TMA) is synthesized by intestinal flora from choline, lecithin, and carnitine, which are primarily derived from dietary sources such as meat, fish, and eggs. Subsequently, TMA is absorbed by the intestines and transported via the portal circulation to the liver, where it undergoes direct conversion to TMAO (Querio et al. 2023). The production of TMAO is influenced by the diversity and composition of the intestinal bacteria, leading to variations in TMAO levels, particularly in conditions of dysbiosis, which are often associated with elevated TMAO concentrations (Schoeler and Caesar 2019). TMAO serves multiple biological functions, including the regulation of cholesterol metabolism, modulation of atherosclerotic processes, and a potential link to an increased risk of cardiovascular disease (Wang and Zhao 2018).

Several studies have indicated that TMAO is involved in the regulation of VEGF expression. Yang et al. (2022) treated HCT-116 cells with TMAO and observed that it enhanced both the proliferation capacity of the cells and the production of VEGF-A. Another study found that TMAO can promote MAPK phosphorylation, thereby enhancing the migration ability of liver cancer cells. Additionally, TMAO can increase the expression of VEGF in liver cancer cells, further influencing the occurrence and development of liver cancer (Zhou et al. 2024).

Furthermore, TMAO also affects tumor progression via other targets. One study discovered that, TMAO activates the ILK/AKT/mTOR signaling pathway through POSTN, leading to increased proliferation, migration, and invasion of Hepa1-6 and Huh7 cells (Wu et al. 2022). Additionally, TMAO can participate in various chronic diseases such as atherosclerosis, thrombosis, hypertension, and chronic kidney disease, potentially becoming a driver for multiple types of tumors. TMAO has also been shown to activate the NF-κB signaling pathway, which promotes the expression of cytokines and adhesion molecules in endothelial cells and vascular smooth muscle cells (VSMCs). The elevated levels of these cytokines and adhesion molecules facilitate the recruitment of leukocytes and contribute to the development of atherosclerosis (Seldin et al. 2016). Similarly, TMAO promotes protein kinase R-like endoplasmic reticulum kinase (PERK)/Akt/mTOR pathway, as well as through NLRP3 and caspase-1 signaling (Kapetanaki et al. 2021). In summary, TMAO exerts a promoting effect in cancer by targeting the VEGF, PI3K, NF-κB, and PERK/Akt/mTOR pathways (Fig. 4).

#### Indole

Indole and its derivatives are intricately linked to the gut microbiome and human health. These compounds can influence gastrointestinal motility, potentially affecting constipation and diarrhea (Su et al. 2022; Liu et al. 2023b). Within the gut environment, gut bacteria can directly utilize dietary tryptophan, converting approximately 4–6% of it into indole and its derivatives, including indole acid derivatives. Furthermore, the gut bacteria can transform tryptophan into various molecules, including ligands for the aryl hydrocarbon receptor (AHR), such as indole and its derivatives: indole aldehyde (IAld), indole acetic acid (IAA), indole propionic acid (IPA), indole ethanal (IAAld), and indole acrylic acid (Agus et al. 2018).

The aromatic hydrocarbon receptor (AHR), a ligand-activated transcription factor involved in the regulation of tumor proliferation, invasion, metastasis, and immune escape, has recently become a star target for cancer therapy (Dai et al. 2022). Some study reported that indole-3-lactic acid, as a ligand for AHR, activates the AHR signaling pathway to inhibit the growth and spread of CRC cells and reduce the incidence of CRC (Wang et al. 2024; Li et al. 2024). Another study found that indoxyl sulfate induced the proliferation of CRC-derived HCT-116 cells by activating the AHR and the proto-oncogene AKT. Moreover, indoxyl sulfate upregulated the expression of the epidermal growth factor receptor (EGFR), increasing the sensitivity of CRC cells to EGF (Ichisaka et al. 2024). These studies suggest that indole derivatives, acting as ligands, can influence AhR and thereby affect the occurrence and development of cancer.

Li et al. (2021b) demonstrated that IPA inhibits the PI3K/AKT/mTOR signaling pathway. Additionally, another study indicated that IAA activates the transcription factors STAT3 and RORγt, which promote IL-17A production by immune cells and enhance the expression of various pro-inflammatory cytokines (Shen et al. 2022). Collectively, these studies suggest that indole derivatives exert their effects on disease by targeting the PI3K/AKT pathway, and STAT3 (Fig. 4).

#### Others

There are additional evidences indicating that gut bacteria -drived metabolites play a significant role in anti-cancer targeted therapy and the cancer progression. Specifically, *F. nucleatum* has been shown to enhance exosome production in gastric cancer cells, which in turn increases HOTTIP expression and facilitates gastric cancer invasion via the miR-885-3p/EphB2/PI3K/AKT signaling pathway (Xin et al. 2023).

Several studies have provided evidence that metabolites produced by specific intestinal bacteria can influence the development of CRC (Bennedsen et al. 2022; Schirbel et al. 2013). Notably, enterotoxin-producing *Enterotoxigenic Bacteroides fragilis* (ETBF) has been found to be enriched in the intestines of CRC patients. This enrichment activates the STAT3 and NF-κB signaling pathways through various virulence factors, which in turn triggers intestinal inflammatory responses and accelerates CRC progression (Wu et al. 2009). Additionally, cell-free supernatants (CFSs) from *Lactobacillus rhamnosus* GG, *Lactobacillus casei* M3, and *Lactobacillus plantarum* YYC-3 have been shown to inhibit the expression and secretion of VEGFA in colorectal cancer cells, which subsequently reduces the expression of MMP2 and MMP9, thereby suppressing the migration and invasion of colorectal cancer cells (Yue et al. 2020). Furthermore, metabolites secreted by *Lactobacillus casei*, *Lactobacillus paracasei*, *Lactobacillus rhamnosus*, and *Lactobacillus plantarum* have been found to inhibit the expression of ErbB-2 and ErbB-3 genes, which subsequently decreases tumor proliferation mediated by these receptors (Faghfoori et al. 2020). Together, these findings suggest that specific metabolites produced by intestinal bacteria can activate critical targets such as NF-κB, PI3K/AKT, and ErbB2/ErbB3, thereby contributing to the development of colorectal cancer.

Recent studies have found that the gut bacteria can also produce gamma-aminobutyric acid (GABA) and regulate the body through neural pathways, blood circulation, and the immune system. GABA plays a significant role in anxiety and depression, tumor suppression, functional bowel diseases, and metabolic disorders (Strandwitz et al. 2019). One study found that GABA significantly inhibited the proliferation of cholangiocarcinoma and the expression of VEGF-A/C, while also promoting the apoptosis of cancer cells (Fava et al. 2005). Other studies have also demonstrated that GABA can reduce the expression of VEGF in lung cancer cells (Banerjee et al. 2015; Al Khashali et al. 2023). These studies indicate that GABA can regulate VEGF expression, thereby influencing tumor progression.

The intestinal bacteria play a pivotal role in cancer development and the effectiveness of targeted therapies. Specific gut bacteria modulate key signaling pathways related to cancer cell proliferation, survival, and migration. For instance, *Listeria monocytogenes* and *F. nucleatum* can activate RTKs like ErbB2, ErbB3, and EGFR, promoting tumor growth and resistance to RTK-targeted therapies in CRC. In contrast, beneficial bacteria such as *Bacillus polyfermentans* have shown antitumor potential by reducing RTK expression and inhibiting cancer cell proliferation. Metabolites derived from intestinal bacteria also influence signaling pathways crucial for cancer progression. LPS, a component of gram-negative bacteria, can activate the NF-κB pathway and upregulate VEGFR-3, promoting cancer metastasis and resistance to therapy. Similarly, secondary bile acids like DCA can activate EGFR and its downstream pathways, accelerating cancer development, while others like UDCA show potential in inhibiting tumor growth through suppression of the PI3K/AKT and EGFR pathways. SCFAs, such as butyrate, can exert anti-tumor effects by inhibiting angiogenesis and modulating immune responses, although their effects may vary depending on the cancer type. TMAO, produced by gut flora from dietary choline, enhances VEGF expression and activates the PI3K/AKT pathway, contributing to tumor growth. Indole derivatives, produced from tryptophan by gut bacteria, modulate the AHR and PI3K/AKT pathways, influencing cancer cell proliferation and immune responses.

These findings suggest that targeting the intestinal bacteria and its metabolites could enhance the efficacy of cancer therapies by modulating key signaling pathways involved in tumorigenesis.

## Impact of intestinal bacteria on anti-cancer-targeted drug resistance

Tumor resistance has many mechanisms, posing a significant challenge in cancer treatment. These include genetic mutations in tumor cells, changes in the tumor microenvironment, reprogramming of signaling pathways, cellular heterogeneity, and drug efflux. Recent studies have also found that the modulation of cell death pathways, including apoptosis, necroptosis, autophagy, ferroptosis, pyroptosis, and necroptosis, has been shown to be a key mechanism in the development of drug resistance in tumor cells. These mechanisms collectively lead to tumor resistance to treatment, affecting therapeutic efficacy. In terms of drug efflux mechanisms, tumors often develop resistance to drug therapy through mechanisms that impede drug access to their sites of action. A prominent mechanism involves the upregulation of members of the ATP-binding cassette (ABC) transporter family, such as P-glycoprotein, MDR1, and BCRP (Thomas and Tampé 2020; Robey et al. 2018).

For targeted therapies and chemotherapeutic drug to be effective, it must penetrate the cell membrane and evade extracellular excretion by efflux transporter proteins (Alam and Locher 2023). Multiple studies have demonstrated that intestinal flora and their metabolites can influence the ABC transporter family of proteins, potentially leading to resistance against targeted drugs in humans. The *Salmonella* nanoparticle mimic, a foodborne pathogen, enters the intestine through the stomach and proliferates within the intestinal environment. Research has identified the *Salmonella* type III secretion effector, SipA, as a key modulator of P-glycoprotein (P-gp) through a pathway involving caspase-3. In this context, Mercado-Lubo developed a nanoparticle model aimed at inhibiting tumor cell growth and overcoming multidrug resistance in tumors by suppressing P-gp expression in tumor cells (Mercado-Lubo et al. 2016).

Additionally, numerous studies have shown that butyrate and secondary bile acids, which are metabolites of intestinal bacteria, significantly influence P-gp activity. Specifically, three secondary bile acids—LCA, DCA, and UDCA—have been shown to enhance both the expression and function of P-gp in vitro (Foley et al. 2021). However, these bile acids alone were insufficient to elicit this effect during dosage testing (Mickley et al. 1989). Additionally, butyrate has been observed to induce P-gp transcription across various cancer models. Notably, butyrate and these three secondary bile acids work synergistically to maximize P-gp expression. The mechanisms by which butyrate and secondary bile acids enhance P-gp expression involve a complex signaling network, which can be summarized at three levels: butyrate and bile acids promote P-gp transcription through HDAC inhibition and nuclear receptor activation [specifically, pregnane X receptor (PXR) and vitamin D receptor (VDR)]. The combination of butyrate and bile acids not only increases the expression of transcription factors that activate P-gp transcription but also decreases the expression of those that inhibit it, ultimately resulting in enhanced P-gp expression (Foley et al. 2021). These metabolites also elevate the expression of transcriptional factors, including Pim-1 kinase, which contribute to the post-translational modifications of P-gp. Research by Alexandra L. Degraeve et al. has indicated that metabolites from intestinal bacteria can modulate the transcription of ABCB1, thereby affecting the pharmacokinetics (PK) of drugs (Degraeve et al. 2023). Consequently, gut bacteria may synergistically enhance P-gp expression through butyrate and secondary bile acids, which could subsequently influence resistance to targeted cancer therapies. Furthermore, it has been established that gut bacteria metabolites can modulate the ABCG2/BCRP transport process, thereby impacting cancer drug resistance (González-Sarrías et al. 2013).

The modulation of cell death pathways, including apoptosis, necroptosis, autophagy, ferroptosis, pyroptosis, and necrosis, has been shown in numerous studies to be a crucial mechanism by which tumor cells develop drug resistance. *F. nucleatum* has been reported to target TLR4, MYD88, and specific microRNAs to activate the autophagy pathway and influence the treatment response in CRC. Consequently, *F. nucleatum* orchestrates a molecular network involving Toll-like receptors, microRNAs, and autophagy, thereby clinically, biologically, and mechanistically regulating drug resistance in CRC (Yu et al. 2017).

*Peptostreptococcus stomatis* can become enriched in the intestines of CRC patients. ERBB2 is an emerging resistance factor for RTK inhibitors in CRC. *P. stomatis* activates the ERBB2-MAPK signaling pathway, thereby promoting the occurrence of colorectal tumors. Furthermore, it can influence the antagonistic effect of EGFR inhibitors on tyrosine kinase receptors and diminish the therapeutic efficacy of BRAF inhibitors, potentially leading to the development of resistance to certain targeted therapies in patients with colorectal cancer (Huang et al. 2024). However, it has been rarely reported that the gut bacteria and its metabolites can influence tumor resistance through other pathways. This could serve as a new research direction for gut bacteria, broadening our understanding of the relationship between gut bacteria and targeted therapies.

In conclusion, intestinal bacteria and their metabolites may influence tumor-associated drug-resistant molecules, leading us to hypothesize that these factors could also affect resistance to targeted therapies.

## Clinical research on FMT in cancer therapy

Fecal microbiota transplantation (FMT) is a therapeutic strategy that entails the transfer of gut microbiota from a healthy donor into a patient’s intestinal tract, aiming to restore the equilibrium of the gut microbiome. In recent years, FMT has gained prominence for its effectiveness in treating a variety of diseases, particularly gastrointestinal disorders such as recurrent *Clostridium difficile* infections, where it has demonstrated considerable success. Furthermore, the potential of FMT in cancer treatment is becoming increasingly apparent. Research indicates that FMT can regulate the gut microbiome, enhance the intestinal immune environment, and bolster the immune system’s capacity to identify and eradicate tumor cells. Additionally, FMT may influence the effectiveness of targeted therapies. By affecting specific microbial communities within the gut, FMT can modulate signaling pathways associated with tumor initiation, proliferation, and metastasis, thereby offering novel therapeutic strategies for cancer patients (Khoruts and Sadowsky 2016).

A randomized clinical trial assessed the efficacy of FMT in treating diarrhea induced by TKIs. The findings indicated that donor FMT was significantly more effective than placebo FMT, with no serious adverse events reported. These results suggest that modulation of gut microbiota may represent a promising therapeutic approach for managing TKI-induced diarrhea. Furthermore, this supports the notion that manipulation of gut microbiota could provide innovative treatment options not only for gastrointestinal disorders but also for drug-induced side effects in cancer patients (Ianiro et al. 2020).

FMT combined with immunotherapy has shown a favorable safety profile and promising antitumor effects in patients with specific cancer types. A clinical study conducted at Peking University revealed that FMT capsules may enhance the efficacy of anti-PD-1 treatment in patients with PD-(L)1-resistant digestive system cancers (NCT04130763) (Peng et al. 2023). In a trial at the University of Pittsburgh, 15 melanoma patients who had previously not responded to treatments underwent FMT, resulting in 6 patients demonstrating a response to the previously ineffective immunotherapy (Davar et al. 2021). Additionally, a trial conducted by Sheba Medical Center in Israel found that 3 out of 10 participants became responders following the transplant (Baruch et al. 2021).

In recent years, FMT has been explored in conjunction with immunotherapy and targeted therapy, demonstrating potential improvements in treatment outcomes. One study illustrated that the combination of FMT with anti-VEGF and anti-PD-1 therapies effectively enhanced treatment for proficient mismatch repair (pMMR) and microsatellite stable (MSS) CRC (Cheng et al. 2023). Additionally, an open-label, single-arm, phase II clinical trial (RENMIN-215) investigated the efficacy of FMT combined with Tislelizumab and Fruquintinib in patients with refractory microsatellite stable metastatic CRC. The findings revealed that this combination therapy not only improved overall survival but also maintained a manageable safety profile, presenting a promising new treatment option for this patient demographic. Notably, the treatment did not alter the structure of the peripheral blood T-cell receptor (TCR) repertoire; however, the expanded TCRs exhibited characteristics indicative of antigen-driven responses among the responders (Zhao et al. 2023). Furthermore, an ongoing clinical trial is assessing the safety and efficacy of FMT in conjunction with Atezolizumab and Bevacizumab for patients with advanced hepatocellular carcinoma (aHCC) who have previously failed immunotherapy (NCT05750030). Another ongoing trial, conducted by the Chinese Academy of Medical Sciences, is evaluating the efficacy and safety of FMT combined with Sintilimab and Fruquintinib as a later-line treatment in patients with advanced-stage CRC (NCT05279677). Overall, FMT has shown promising potential in enhancing the efficacy of targeted therapies and immunotherapies in cancer treatment, with several clinical trials indicating improved outcomes across various cancer types. The combination of FMT with therapies such as anti-PD-1, anti-VEGF, and Regorafenib has yielded encouraging results, suggesting that modulation of gut microbiota may represent a novel strategy to enhance cancer treatment responses (Table 2).

**Table 2:** Clinical research overview of FMT in cancer treatment

Cancer type	Drug	Results	References
mRCC	TKI	Donor FMT is more effective than placebo FMT in treating TKI-induced diarrhea, with no serious adverse events observed	NCT04040712
CRC	Anti-PD-1	FMT combined with anti-PD-1 improves immune response against tumors, especially in PD-(L)1-resistant patients	Peking University (NCT04130763)
Melanoma	Anti-PD-1	15 melanoma patients who failed previous treatments received FMT, and 6 showed a response to previously ineffective immunotherapy	University of Pittsburgh
CRC	Anti-VEGF and anti-PD-1	FMT combined with anti-VEGF and anti-PD-1 improves treatment outcomes in pMMR MSS stage IVB CRC patients	Yunnan Tumour Hospital Kunming Medical University No.3 Affiliated Hospital
mCRC	Tislelizumab and fruquintinib	FMT, Tislelizumab, and Fruquintinib combination significantly improved survival and showed manageable safety in refractory microsatellite stable mCRC patients	RENMIN-215
HCC	Atezolizumab and bevacizumab	Ongoing	NCT05750030
CRC	Sintilimab and fruquintinib	Ongoing	NCT05279677

Despite its potential, the application of FMT in cancer treatment encounters several significant challenges. Key obstacles include the rigorous recruitment and selection processes for donors. Additionally, the inherent instability of human feces as a therapeutic component complicates clinicians’ ability to ascertain whether a donor possesses the requisite microbiota and to identify the specific bacterial strains needed by the recipient. Nonetheless, FMT presents a novel approach for enhancing the survival of cancer patients through the modulation of the gut microbiome. As research progresses and technology evolves, the prospects for integrating gut microbiota into cancer therapy appear increasingly promising.

## Conclusion and prospect

Targeted therapies, which specifically focus on molecular markers in cancer cells, have significantly improved treatment efficacy while substantially reducing the side effects associated with conventional chemotherapy, thus enhancing the quality of patient survival (Wang et al. 2023). However, the impact of intestinal bacteria on this process is critical, as they play a vital role in the effectiveness of cancer treatments through various mechanisms. These mechanisms include influencing drug metabolic pathways (Bennedsen et al. 2022), regulating the immune system (Jiao et al. 2020; Overacre-Delgoffe et al. 2021), and modifying the tumor microenvironment (Wong-Rolle et al. 2021). In the context of targeted therapies, alterations in intestinal bacteria can lead to treatment-related adverse effects, such as diarrhea (Su et al. 2021), and may also affect patient responses to treatment and the development of drug resistance by altering the composition and function of the intestinal microbiota (Su et al. 2021; Inukai et al. 2023). Therefore, understanding and leveraging changes in gut bacteria could provide novel strategies for enhancing cancer treatment outcomes, alleviating treatment-related side effects, and overcoming drug resistance. The paper further emphasizes that intestinal bacteria and their metabolites can directly or indirectly regulate several signaling pathways relevant to cancer therapy, including VEGFR, EGFR, and HER2, thereby influencing cancer growth, metastasis, and therapeutic responses (Yu et al. 2020; Tao et al. 2019). Additionally, intestinal bacteria may modulate drug absorption and metabolism, which subsequently affects therapeutic efficacy, by regulating the expression of drug transporter proteins such as P-glycoprotein and BCRPs. Future research may investigate how methods such as FMT can be integrated with targeted therapies to optimize cancer treatment outcomes, overcome drug resistance, and alleviate treatment-related side effects. These advancements are anticipated to provide new hope to cancer patients and offer innovative strategies for cancer prevention, early diagnosis, and treatment.

As the role of the gut microbiome in cancer therapy receives increasing attention, it is essential to consider the microbiomes from other body sites, which may also significantly contribute to tumor initiation and progression. Research indicates that microbiomes in areas such as the oral cavity, skin, and lungs can influence tumor processes through interactions with the gut microbiome, as well as directly affecting tumor biology. For instance, dysbiosis in the oral microbiome is closely linked to the development of oral cancer (Baker et al. 2024), while the skin microbiota may impact the onset and treatment response of skin cancer (Woo et al. 2022). Additionally, pulmonary microbiota may influence lung cancer development by modulating immune responses or through alterations in the microbiome (Zhao et al. 2021). Consequently, investigating the roles of microbiomes from these other body sites and understanding their potential effects on cancer could yield new insights into cancer treatment. Furthermore, tumor-resident microbiota can directly affect tumor initiation and progression. Growing evidence suggests that these microbiota can colonize tumor tissue via mucosal disruption, migration to adjacent tissues, and hematopoietic invasion, thereby influencing tumor biology as a crucial component of the tumor microenvironment. Mechanistic studies indicate that tumor-resident microbiota may facilitate tumorigenesis and progression by inducing genomic instability and mutations, influencing epigenetic modifications, promoting inflammation, evading immune destruction, regulating metabolism, and activating invasion and metastasis (Cao et al. 2024). Therefore, a comprehensive understanding of the interactions between gut bacteria and microbiota from other body sites is vital for elucidating the mechanisms of cancer initiation and treatment, potentially leading to new therapeutic strategies.

## Data Availability

No datasets were generated or analysed during the current study.
